# Single, Dual, and Poly Use of Flavored Tobacco Products Among Youths

**DOI:** 10.5888/pcd15.170389

**Published:** 2018-06-28

**Authors:** Hongying Dai

## Abstract

**Introduction:**

Flavoring has become the leading reason for current tobacco use among adolescents. This study sought to evaluate patterns of flavored tobacco product use and associated risk factors among youths.

**Methods:**

Weighted estimates of single, dual, and poly use of flavored tobacco products were calculated from the 2014 National Youth Tobacco Survey (n = 21,926). Multinomial logistic regression was performed to assess factors associated with flavored product use.

**Results:**

Among current tobacco users (n = 3,805), 70.0% of students were current users of flavored tobacco products: 42.6% used a single flavored product, 16.8% used 2 flavored products (dual users), and 10.6% used more than 2 flavored products (poly users). Flavored product use, especially dual and poly use, was higher among high school students compared with middle school students. Compared with single flavored tobacco product users (36%), dual (57%) and poly users (79%) of flavored tobacco products had higher prevalences of using flavored e-cigarettes (*P* < .001). Non-Hispanic blacks and those of other races had lower prevalences than non-Hispanic whites of using flavored products but not nonflavored products. Tobacco use by household members, no perception of harm from tobacco products, and more frequent exposure to tobacco advertisement in newspapers/magazines and stores were associated with increased odds of flavored product use.

**Conclusion:**

The concurrent use of flavored tobacco products is prevalent among youths. E-cigarettes were the leading flavored product and often concurrently used with other flavored tobacco products. Comprehensive control and prevention strategies to reduce flavored tobacco use among youths are needed.

## Introduction

As cigarette use among youths has been decreasing, tobacco industries have been using flavored tobacco products to attract a new generation of young users (1). Use of flavored tobacco products is prevalent among youths and young adults in the United States (2,3). In 2014, 70% of adolescent tobacco users reported using at least 1 flavored tobacco product in the past 30 days, which represents 3.26 million middle school and high school students (2). Flavored tobacco, including menthol cigarettes and flavored noncigarette tobacco products, could serve as a starter kit for smoking because adolescents often experiment with smoking in pursuit of curiosity and novelty (4). Flavoring has become the leading reason for current tobacco use among teenagers aged 12 to 17 years with 81% of e-cigarette users, 79% of hookah users, 74% of cigar users, 69% of smokeless tobacco users, and 67% of snus users attributing the availability of appealing flavors for their tobacco use in 2013–2014 (5).

The 2009 Family Smoking Prevention and Tobacco Control Act (FSPTCA) banned cigarettes with characterizing flavors (eg, candy, fruit, clove) except menthol. However, there are no restrictions on the marketing and sales of flavored noncigarette tobacco products (6). This has led to a proliferation of flavored tobacco products in the marketplace. For example, there are more than 460 brands and 7,700 flavors of e-cigarettes in the market, such as candy crush, gummy bears, and bubble gum (7).

Tobacco companies spend approximately $10 billion a year in marketing to promote their products (8). Tobacco advertising and promotion can effectively entice youths to smoke by increasing curiosity, fostering positive attitudes toward tobacco use, and using celebrity effects (9). Studies also suggest that exposure to e-cigarette advisements was associated with an increased risk of e-cigarette use (10,11). Little is known about how exposure to tobacco advertising and channels of exposure (ie, the internet, newspapers, stores, and TV) are associated with flavored product use. Other factors, including household member smoking and sociodemographic status, could also be associated with flavored product use among youths (10–13). Understanding the effects of these factors to flavored tobacco use is needed to formulate strategies and interventions in reducing flavored tobacco use among youths. Existing studies among youths have reported the prevalence of using a single flavored tobacco product (2,5) and dual use of flavored tobacco products (ie, using 2 flavored products such as flavored little cigars and menthol cigarettes) (14). However, no study has evaluated dual or poly (more than 2 products) use patterns across a range of flavored tobacco products among youths. Youths using multiple tobacco products could become addicted to nicotine and susceptible to other substance abuse (ie, alcohol, marijuana, and illicit drug use disorders) (15,16). Therefore, it is important to understand use patterns (ie, single, dual, and poly use) of various flavored tobacco products among youths.

To fill the knowledge gaps, this study used data from the 2014 National Youth Tobacco Survey (NYTS) to analyze the single, dual, and poly use patterns of 8 flavored tobacco products among middle school and high school students and to further examine the factors that could be associated with flavored tobacco product use.

## Methods

### Data

The 2014 NYTS is a cross-sectional and school-based annual survey, covering tobacco-related knowledge, attitudes, and behaviors of middle school (grades 6-8) and high school (grades 9-12) students in the United States. The 2014 NYTS was conducted by using a stratified, 3-stage cluster sampling procedure. A detailed description of the 2014 NYTS survey design, questionnaires, and data collection process can be found on the NYTS website (17). In 2014, a total of 22,007 students from 207 schools completed the NYTS questionnaire. The school response rate was 80.2% and the student response rate was 91.4%, yielding an overall response rate of 73.3% (18). Because NYTS provides public data with de-identified information, this study is treated as not a human subjects study by the institutional review board of Children’s Mercy Hospital.

### Measures

#### Current use of flavored tobacco products

Current use of any tobacco is defined by use of cigarettes, cigars (including cigars, cigarillos, and little cigars), smokeless tobacco (including chewing tobacco, snuff, and dip), e-cigarettes, hookahs, tobacco pipes, snus, or dissolvables at least 1 day in the last 30 days. This study excluded from analysis 81 students whose answers were missing or inconsistent, resulting in 21,926 respondents in the study.

Because youths tended to underreport menthol use status from brands that predominantly produce menthol cigarettes (eg, Newport, Kool) (19), this study followed the same approach from a previous study (2) to define menthol cigarette users based on 2 items: 1) “During the past 30 days, what brand of cigarettes did you usually smoke? (CHOOSE ONLY ONE ANSWER)” and 2) “Menthol cigarettes are cigarettes that taste like mint. During the past 30 days, were the cigarettes that you usually smoked menthol?” Those who reported Kool or Newport as the usual cigarette brand or those who reported yes to the menthol question were classified as menthol cigarette users. Of 763 current menthol cigarette users, 628 (82.3%) responded yes to usually smoking menthol cigarettes, 7 (1%) reported using Kool, and 128 (16.7%) reported using Newport.

Flavored noncigarette tobacco product use was defined by the question “Which of the following tobacco products that you used in the past 30 days were flavored to taste like menthol (mint), alcohol (wine, cognac), candy, fruit, chocolate or other sweets? (CHOOSE ALL THAT APPLY)” Responses to this question were “Cigars, cigarillos, or little cigars,” “chewing tobacco, snuff, or dip,” “Electronic cigarettes, or e-cigarettes,” “Smoking tobacco out of a hookah or waterpipe,” “Pipe filled with tobacco (not waterpipe),” “Snus,” “Dissolvable tobacco products,” and “I didn’t use flavored tobacco products in the past 30 days.” Those who selected at least 1 flavored product were categorized as flavored noncigarette product users.

Students who reported use of any flavored noncigarette tobacco product or menthol cigarettes were categorized as flavored-product users. Those who reported current use of any tobacco product but did not select a flavored product were categorized as nonflavored-product users. Those who used only 1 flavored product were classified as single flavored-product users (not counting the nonflavored-product use). Those who concurrently used 2 flavored products were classified as dual flavored-product users, and those who concurrently used more than 2 flavored products were classified as poly flavored-product users. For the dual and poly flavored-product users, this study further separated them based on whether they used e-cigarettes or not.

#### Covariates

Several covariates were included in the analysis based on previous studies (10–13), such as sex (male or female), race/ethnicity (non-Hispanic white, non-Hispanic black, Hispanic, or non-Hispanic other) and grade (middle school or high school). Societal and attitudinal factors have been associated with youth tobacco use, such as tobacco use by household members, perception of tobacco’s danger, and exposure to tobacco’s advertising (10,20), thus these variables were also included in the analysis.

Tobacco use by other household members was defined as single-product use, dual-product use, and poly-product use by using the question “Does anyone who lives with you now . . .? (CHOOSE ALL THAT APPLY)” with the following response options: “Smoke cigarettes,” “Smoke cigars, cigarillos, or little cigars,” “Use chewing tobacco, snuff, or dip,” “Use electronic cigarettes or e-cigarettes,” “Smoke tobacco from a hookah or waterpipe,” “Smoke pipes filled with tobacco (not waterpipes),” “Use snus,” “Use dissolvable tobacco products,” “Smoke bidis (small brown cigarettes wrapped in a leaf),” and “No one who lives with me now uses any form of tobacco.” The perception of tobacco’s danger was measured by the item: “How strongly do you agree with the statement ‘All tobacco products are dangerous’?” The respondents who answered strongly agree or agree were classified into the group with “yes” and those who answered disagree or strongly disagree were classified into the group with “no” regarding perceptions of harm associated with tobacco use.

Self-reported exposure to tobacco advertising was measured by 4 items: “When you are using the Internet, how often do you see ads or promotions for cigarettes or other tobacco products?” “When you read newspapers or magazines, how often do you see ads or promotions for cigarettes or other tobacco products?” “When you go to a convenience store, supermarket, or gas station, how often do you see ads or promotions for cigarettes or other tobacco products?” and “When you watch TV or go to the movies, how often do you see actors and actresses using cigarettes or other tobacco products?” Four variables were created to measure the channels of exposure to tobacco advertising: the internet, newspapers/magazines, stores, and TV/movies. Response options ranging from “never,” “rarely,” “sometimes,” “most of the time,” to “always” were treated as ordinal variables and coded as 0 (“never”) to 4 (“always”). When participants did not respond to survey questions or participants responded “I don’t know,” the corresponding covariates were set as missing values.

### Statistical methods

Weighted estimates along with 95% confidence intervals (CIs) of flavored tobacco use patterns were calculated, both overall and by middle school and high school student status. Sampling weights and survey stratum were included in the analysis to account for the complex survey design. In univariate analysis, Rao-Scott χ^2^ test was performed to compare the distribution of flavored tobacco product use by sociodemographic factors. A multinomial logistic regression model was used to examine the associations between use patterns of flavored tobacco products and demographic characteristics (sex, race/ethnicity and grade), social and attitudinal factors (tobacco use by household members and perception of tobacco’s danger), and exposure to tobacco advertisements (internet, newspaper/magazine, store, TV/movie). Students with no use of any tobacco product in the past 30 days served as the control group. Adjusted odds ratios (AOR) and 95% CIs were calculated in the multivariable logistic analysis. Statistical analyses were performed by using SAS 9.4 (SAS Institute, Inc), and a *P* value less than .05 was considered significant.

## Results

Of all respondents (n = 21,926), 3,805 (weighted percentage, 17.1%) were current tobacco users. Among current tobacco users, 2,638 (weighted percentage, 70.0%) were current users of flavored tobacco product(s) (Figure); 1,666 (weighted percentage, 42.6%) single flavored-product users, 601 (weighted percentage, 16.8%) dual flavored-product users, and 371 (weighted percentage, 10.6%) poly flavored-product users. Compared with single flavored-product users (36%), dual users (57%) and poly users (79%) of flavored products had a higher prevalence of using flavored e-cigarettes (*P* < .001).

**Figure:**
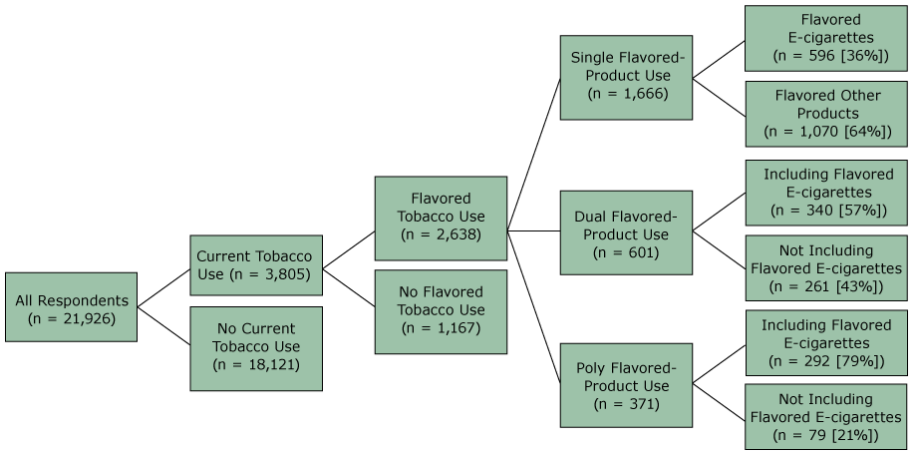
Flavored tobacco product use among US middle school and high school students, National Youth Tobacco Survey, 2014. Single flavored-product use was defined as using only 1 flavored tobacco product; dual flavored-product use was defined as using 2 flavored tobacco products, and poly flavored-product use was defined as using more than 2 flavored tobacco products.

### Use patterns

The proportion of current use of flavored products was higher among high school students compared with middle school students (73.0% vs 57.0%, *P* < .001) (Table 1). Among single-flavored-product users, e-cigarettes (15.9%), hookahs (9.2%), and menthol cigarettes (6.0%) were the most popular tobacco products. The most common dual flavored-product use combinations were e-cigarettes/hookahs (3.7%) and e-cigarettes/menthol cigarettes (2.9%).

**Table 1:** Patterns of Flavored Tobacco Product Use^a^ Among Current Tobacco Users, National Youth Tobacco Survey, 2014^b^

Characteristic	Overall		School Type			
			Middle School		High School	
	Unweighted N^c^	Weighted % (95% CI)	Unweighted N	Weighted % (95% CI)	Unweighted N	Weighted % (95% CI)
**Current tobacco use**	3,805	100	873	100	2,889	100
Nonflavored	1,167	30.0 (27.5–32.6)	363	43.0 (35.8–50.2)	794	27.0 (24.6–29.4)
Flavored	2,638	70.0 (67.4–72.5)	510	57.0 (49.8–64.2)	2,095	73.0 (70.6–75.4)
**Single flavored-product use**	1,666	42.6 (40.6–44.7)	364	39.9 (34.3–45.5)	1,284	43.3 (41.0–45.6)
E-cigarettes	596	15.9 (13.1–18.6)	166	18.3 (14.1–22.5)	424	15.4 (12.2–18.5)
Hookah	363	9.2 (7.0–11.3)	62	6.2 (4.0–8.4)	297	9.9 (7.3–12.4)
Menthol cigarettes	263	6.0 (4.8–7.2)	53	5.9 (4.0–7.9)	204	5.9 (4.5–7.3)
Cigar	227	5.7 (4.6–6.8)	38	4.5 (2.6–6.4)	188	6.0 (4.7–7.3)
Smokeless	199	5.6 (4.1–7.1)	37	4.4 (1.9–7.0)	161	5.9 (4.2–7.6)
Other	18	0.4 (0.2–0.5)	8	0.6 (0.1–1.1)^d^	10	0.3 (0.1–0.5)
**Dual flavored-product use**	601	16.8 (15.3–18.3)	96	10.9 (8.0–13.9)	493	18.1 (16.3–19.9)
Including e-cigarette	340	9.9 (8.3–11.5)	53	5.4 (3.4–7.4)	279	10.9 (9.0–12.7)
E-cigarettes and hookah	134	3.7 (2.8–4.7)	19	1.8 (0.5–3.1)^d^	111	4.1 (3.0–5.2)
E-cigarettes and menthol cigarette	99	2.9 (2.1–3.7)	19	1.9 (1.0–2.8)	80	3.2 (2.2–4.1)
E-cigarettes and cigar	68	2.2 (1.3–3.1)	9	1.3 (0.3–2.2)^d^	57	2.4 (1.3–3.5)
E-cigarettes and other	39	1.1 (0.6–1.6)	6	0.4 (0.0–0.9)^d^	31	1.2 (0.6–1.8)
Not including e-cigarettes	261	6.9 (5.4–8.5)	43	5.5 (3.0–8.1)	214	7.2 (5.5–8.9)
Menthol cigarettes and cigar	77	1.9 (1.1–2.6)	17	2.6 (0.3–4.9)^d^	58	1.7 (1.0–2.3)
Hookah and cigar	40	1.1 (0.8–1.5)	2	0.2 (0.0–0.5)^d^	37	1.4 (0.9–1.8)
Other	144	3.9 (2.9–4.8)	24	2.8 (1.3–4.2)	119	4.2 (3.0–5.4)
**Poly flavored-product use**	371	10.6 (8.9–12.2)	50	6.2 (3.4–8.9)	318	11.6 (9.9–13.3)
Including e-cigarettes	292	8.2 (6.6–9.7)	41	4.3 (2.4–6.3)	248	9.1 (7.3–10.8)
Not including e-cigarettes	79	2.4 (1.5–3.2)	9	1.8 (0.0–4.2)^d^	70	2.5 (1.7–3.4)

Abbreviation: CI, confidence interval.

a Single flavored-product use was defined as using only 1 flavored tobacco product; dual flavored-product use was defined as using 2 flavored tobacco products, and poly flavored-product use was defined as using more than 2 flavored tobacco products.

b The analysis took sampling weights and survey strata into account. The 8 flavored tobacco products were e-cigarettes, hookah, menthol cigarettes, cigars (including cigars, cigarillos and little cigars), smokeless tobacco (including chewing tobacco, snuff, and dip), pipes, snus, and dissolvables.

c Numbers may not equal totals because of missing data.

d Relative standard error >30%, indicating data could be statistically unstable.

Among current tobacco users, exclusive use of flavored e-cigarettes was 18.3% among middle school students and 15.4% among high school students. A greater percentage of high school current tobacco users (9.9%) than middle school users (6.2%) were exclusive users of flavored hookahs, but a similar percentage of middle school and high school current tobacco users were exclusive users of menthol cigarettes. High school current tobacco users were more likely than middle school users to be dual or poly users of flavored products (*P* < .001).

Overall, a smaller percentage of younger current tobacco users (≤15 years old) than older tobacco users (>15 years old) reported flavored tobacco use (59.9% vs 72.9%, *P* < .001) (Table 2). A similar proportion of male and female current tobacco users (69.5% vs. 70.4%, *P* = .63) reported flavored tobacco use. A smaller proportion of non-Hispanic black current tobacco users reported using flavored tobacco products compared with non-Hispanic whites (55.6% vs 75.1%, *P* < .001).

**Table 2:** Prevalence of Flavored Tobacco Product^a^ Use^b^ by Demographic Factors, National Youth Tobacco Survey, 2014 (n = 3,805)^c^

Characteristic	Any Flavored Product Use	Single Flavored-Product Use		Dual Flavored-Product Use		Poly Flavored-Product Use	
		E-cigarettes	Others	Including E-cigarettes	Not Including E-cigarettes	Including E-cigarettes	Not Including E-cigarettes
**Unweighted N**	2,638	596	1,070	340	261	292	79
**Weighted N (in 10,000s)**	326	74	125	46	32	38	11
**Proportion of flavored use among current tobacco users**	70.0 (67.4–72.5)	15.9 (13.1–18.6)	26.7 (23.6–29.8)	9.9 (8.3–11.5)	6.9 (5.4–8.5)	8.2 (6.6–9.7)	2.4 (1.5–3.2)
**Age, y^d^, weighted % (95% confidence interval)**							
<15	59.9 (53.6–66.2)	19.1 (14.7–23.5)	23.5 (19.2–27.8)	6.5 (4.6–8.4)	4.9 (2.8–7.0)	3.9 (2.3–5.5)	2.0 (0.0–4.0)^e^
≥15	72.9 (70.4–75.4)	15.0 (12.0–17.9)	27.7 (24.1–31.4)	10.8 (8.9–12.7)	7.5 (5.8–9.2)	9.4 (7.6–11.2)	2.5 (1.6–3.3)
**Sex^d^, weighted % (95% confidence interval)**							
Male	69.5 (66.7–72.3)	14.4 (11.8–17.1)	26.8 (23.4–30.1)	8.0 (6.0–10.0)	7.9 (6.0–9.8)	9.3 (7.1–11.6)	3.1 (1.7–4.5)
Female	70.4 (66.9–74.0)	18.0 (14.2–21.8)	26.7 (22.5–30.9)	12.1 (10.2–14.1)	5.7 (4.1–7.3)	6.5 (4.6–8.3)	1.5 (0.6–2.4)
**Race/ethnicity^d^, weighted % (95% confidence interval)**							
Non-Hispanic white	75.1 (72.7–77.5)	17.0 (13.4–20.7)	25.5 (21.6–29.4)	11.0 (8.9–13.1)	7.9 (5.5–10.2)	10.4 (8.1–12.7)	3.3 (2.0–4.7)
Non-Hispanic black	55.6 (49.9–61.3)	9.7 (5.2–14.1)	30.8 (26.6–35.1)	4.0 (1.4–6.5)^e^	6.7 (4.8–8.6)	3.2 (1.2–5.1)^e^	1.3 (0.2–2.4)^e^
Hispanic	66.1 (61.4–70.8)	16.4 (12.5–20.3)	28.0 (22.4–33.6)	9.9 (7.4–12.5)	5.2 (3.8–6.7)	5.5 (3.7–7.4)	1.0 (0.2–1.7)^e^
Non-Hispanic other	65.5 (58.0–73.0)	18.5 (12.5–24.4)	26.7 (20.2–33.2)	9.0 (2.8–15.2)^e^	5.4 (2.3–8.5)	5.5 (2.1–8.9)^e^	0.4 (0.0–1.1)^e^
**School type^d^, weighted % (95% confidence interval)**							
Middle school	57.0 (49.8–64.2)	18.3 (14.1–22.5)	21.6 (17.1–26.2)	5.4 (3.4–7.4)	5.5 (3.0–8.1)	4.3 (2.4–6.3)	1.8 (0.0–4.2)^e^
High school	73.0 (70.6–75.4)	15.4 (12.2–18.5)	27.9 (24.3–31.6)	10.9 (9.0–12.7)	7.2 (5.5–8.9)	9.1 (7.3–10.8)	2.5 (1.7–3.4)

a The 8 flavored tobacco products are e-cigarettes, hookah, menthol cigarettes, cigars (including cigars, cigarillos, and little cigars), smokeless tobacco (including chewing tobacco, snuff, and dip), pipes, snus, and dissolvables.

b Single flavored-product use was defined as using only 1 flavored tobacco product; dual flavored-product use was defined as using 2 flavored tobacco products, and poly flavored-product use was defined as using more than 2 flavored tobacco products.

c The analysis took sampling weights and survey strata into account. Weighted N is an estimate of US youth users of (flavored) tobacco product(s).

d Rao-Scott χ^2^ test was performed to compare with distribution of any flavored product use by age, sex, race/ethnicity, and school type. *P* < .001 for age, race/ethnicity, and school type; *P* = .63 for sex.

e Relative standard error >30%, indicating data could be statistically unstable.

### Factors associated with flavored product use

Boys had higher odds of reporting current use of both flavored and nonflavored tobacco products than girls (Table 3). High school (vs middle school) students had higher odds of reporting nonflavored product use (AOR, 2.6; 95% CI, 1.9–3.5), single flavored-product use (AOR, 4.9; 95% CI, 3.9–6.1), dual flavored-product use (AOR, 7.0; 95% CI, 4.9–9.9), and poly flavored-product use (AOR, 7.6; 95% CI, 4.5–12.9). Non-Hispanic blacks and other races were less likely than non-Hispanic whites to report use of flavored products but not nonflavored products, while Hispanics were more likely than non-Hispanic whites to report use of nonflavored and single flavored products.

**Table 3:** Multinomial Logistic Regression for Factors Associated With Nonflavored Tobacco Product Use and Single, Dual, and Poly Flavored-Product Use,^a^ National Youth Tobacco Survey, 2014

Characteristic	Nonflavored-Product Use		Single Flavored-Product Use		Dual Flavored-Product Use		Poly Flavored-Product Use	
	AOR^b^ (95% CI)	*P* Value	AOR^b^ (95% CI)	*P* Value	AOR^b^ (95% CI)	*P* Value	AOR^b^ (95% CI)	*P* Value
**Sex**								
Male	1.5 (1.2–1.8)	<.001	1.3 (1.2–1.5)	<.001	1.4 (1.2–1.7)	<.001	2.3 (1.5–3.5)	<.001
Female	1 [Reference]							
**School type^c^**								
Middle school	1 [Reference]							
High school	2.6 (1.9–3.5)	<.001	4.9 (3.9–6.1)	<.001	7.0 (4.9–9.9)	<.001	7.6 (4.5–12.9)	<.001
**Race**								
Non-Hispanic white	1 [Reference]							
Non-Hispanic black	1.1 (0.9–1.4)	0.35	0.6 (0.5–0.8)	<.001	0.4 (0.3–0.7)	<.001	0.3 (0.2–0.5)	<.001
Hispanic	1.7 (1.4–2.1)	<.001	1.4 (1.2–1.8)	<.001	1.2 (0.8–1.6)	.42	0.7 (0.4–1.1)	.12
Non-Hispanic other	0.8 (0.6–1.1)	0.24	0.7 (0.5–1.0)	.04	0.5 (0.3–1.0)	.06	0.3 (0.2–0.6)	<.001
**Tobacco use by household members**								
None	1 [Reference]							
Single flavored-product use	2.3 (1.9–2.9)	<.001	1.9 (1.7–2.2)	<.001	1.7 (1.2–2.3)	.001	2.0 (1.3–3.0)	.001
Dual flavored-product use	2.8 (2.1–3.7)	<.001	3.5 (2.8–4.3)	<.001	4.1 (3.1–5.3)	<.001	4.6 (3.2–6.6)	<.001
Poly flavored-product use	4.9 (3.5–6.8)	<.001	6.0 (4.6–7.7)	<.001	9.5 (6.3–14.4)	<.001	22.6 (14.7–34.7)	<.001
**Agree that “all tobacco products are dangerous”**								
No	3.0 (2.4–3.9)	<.001	3.3 (2.8–4.0)	<.001	3.7 (2.8–4.9)	<.001	6.2 (4.4–8.7)	<.001
Yes	1 [Reference]							
**Exposure to tobacco advertisements**								
Internet	1.1 (1.0–1.2)	.21	1.0 (0.9–1.1)	.81	0.9 (0.7–1.0)	.07	0.9 (0.8–1.1)	.29
Newspaper/magazine	1.1 (1.0–1.2)	.06	1.1 (1.0–1.2)	.02	1.1 (1.0–1.3)	.03	1.1 (1.0–1.3)	.05
Store	0.9 (0.9–1.0)	.05	1.1 (1.0–1.1)	.07	1.2 (1.0–1.3)	.01	1.5 (1.2–1.7)	<.001
TV/movie	0.9 (0.9–1.0)	.20	0.9 (0.9–1.0)	.11	1.0 (0.9–1.2)	.93	0.9 (0.7–1.0)	.05

Abbreviations: AOR, adjusted odds ratio; CI, confidence interval.

a Single flavored-product use was defined as using only 1 flavored tobacco product; dual flavored-product use was defined as using 2 flavored tobacco products, and poly flavored-product use was defined as using more than 2 flavored tobacco products.

b AORs for nonflavored and single, dual, and poly flavored-product use are in reference to no tobacco use.

c Because grade and age are highly correlated, age is not included in the multivariable analysis.

Household tobacco use patterns (single, dual, and poly flavored-product use versus no tobacco use) as well as perceptions of harm associated with tobacco use (no versus yes) were associated with high odds of tobacco use (flavored and nonflavored). More frequent exposure to tobacco advertisements in newspapers/magazines was associated with higher odds of single flavored-product use (AOR, 1.1; 95% CI, 1.0–1.2) and dual flavored-product use (AOR, 1.1; 95% CI, 1.0–1.3), while more frequent exposure to tobacco advertisements in stores was associated with higher odds of dual flavored-product use (AOR, 1.2; 95% CI, 1.0–1.3) and poly flavored-product use (AOR, 1.5; 95% CI, 1.2–1.7).

## Discussion

Past studies (2,5) have evaluated single use of flavored tobacco products and identified that flavored e-cigarette usage surges among youths. But previous studies have not examined concurrent use of various flavored tobacco products. Various flavored tobacco products are available in the market (7) and nicotine levels vary substantially across these products. Studies have suggested that tobacco companies may use flavored products with low nicotine levels to lure new users (1). Because the Food and Drug Administration (FDA) announced that it would seek inputs from the public on approaches to regulate kid-appealing flavors in e-cigarettes and cigars (21), this study adds to the literature by reporting the patterns of flavored tobacco use. The most common dual flavored-product use combinations were e-cigarettes/hookahs and e-cigarettes/menthol cigarettes. Furthermore, most (77%) of poly flavored-product uses included e-cigarettes. E-cigarettes are quickly gaining popularity among youths and dual use of e-cigarettes and other tobacco products could increase risk of addiction to nicotine in this vulnerable population (22).

Further analyses indicated that youths with more frequent exposure to tobacco advertisements in newspapers/magazines and stores were more likely to be flavored tobacco users. The Federal Trade Commission (FTC) reports show that the tobacco industry spends more than 90% of their total marketing budget each year to promote products in convenience stores, gas stations, and other retail outlets (8). This marketing strategy can effectively entice youths, because stores are places that adolescents frequently visit (8). As the demand for cigarettes decreases, tobacco companies have substantially increased marketing of smokeless tobacco, flavored tobacco, and e-cigarettes. For instance, expenditures on e-cigarette advertising increased from $6.4 million in 2011 to $115 million in 2014, which led to a rise of exposure to e-cigarette advertising among youths (23). Flavorings have become the major themes of marketing campaigns for various noncigarette tobacco products, including flavored hookahs and flavored cigars (24,25). The advertisements and packaging use stylish designs and bright colors to emphasize that flavored tobacco products taste like candies, sweetened beverages, and alcohol (26). Exposure to e-cigarette and other tobacco advertisements can increase the risk of smoking initiation and reduce intention to quit (10,11,23). Regulations on advertisements and promotions of flavored tobacco products to youths are warranted.

Concerning social and demographic risk factors, single, dual, and poly use of tobacco products by family members were associated with youth tobacco product use. Parental and sibling smoking is a significant factor associated with smoking behaviors among children (27). E-cigarette use by household members also significantly increased the risk of e-cigarette use among youths (10). Youths often model behaviors of significant others and form attitudes and risk perceptions of smoking by observing household members (27). Smoking by family members can increase access to tobacco products, normalize smoking behaviors, and reduce perceived risks of smoking. As a result, youths living in a household with tobacco use had increased risk of using both flavored and nonflavored tobacco products. Studies have assessed sociodemographic risk factors for single use of flavored tobacco products (1). This study further identified the heterogeneity between nonflavored and flavored tobacco use. Tailored educational campaigns and prevention programs can be developed for these priority youth populations with high risk of using flavored tobacco products.

This study has some limitations. First, the 2014 NYTS data are cross-sectional and the causal inference cannot be established. Second, both flavored tobacco product use and exposure to tobacco advertisements were self-reported, thus they are subject to recall biases, especially for younger respondents (28). Third, flavored tobacco use was measured by a binary variable to indicate whether respondents used any flavored tobacco product in the past 30 days. Additional survey items to quantify the frequency of flavored tobacco use are needed. Fourth, a check-all-that-apply response was used in the 2014 NYTS to ascertain flavored product use. This method might yield lower estimates than forced-choice options (29). Finally, the 2014 NYTS is a school-based survey collected from students who attended either public or private schools. The results might not be generalizable to all school-aged youths.

Despite these limitations, this study contributes to existing literature by identifying a proliferation of dual and poly use of flavored tobacco products among youths, with flavored e-cigarettes as the most common product used. Comprehensive tobacco control policies and prevention strategies, including regulation of marketing, sales, and distribution of flavored tobacco products, smoke-free house rules, and education on harms of all tobacco use, are warranted to reduce flavored tobacco product use among youths. Studies are also needed to compare nicotine concentration level and nicotine dependence among single, dual, and poly flavored-tobacco product users.
